# CD27 expression discriminates porcine T helper cells with functionally distinct properties

**DOI:** 10.1186/1297-9716-44-18

**Published:** 2013-03-11

**Authors:** Katharina Reutner, Judith Leitner, Andrea Müllebner, Andrea Ladinig, Sabine E Essler, J Catharina Duvigneau, Mathias Ritzmann, Peter Steinberger, Armin Saalmüller, Wilhelm Gerner

**Affiliations:** 1 Institute of Immunology, Department of Pathobiology, University of Veterinary Medicine Vienna, Veterinärplatz 1, Vienna 1210, Austria; 2 Institute of Immunology, Center for Pathophysiology, Infectiology and Immunology, Medical University of Vienna, Borschkegasse 8a, Vienna 1090, Austria; 3 Institute for Medical Biochemistry, Department of Biomedical Sciences, University of Veterinary Medicine Vienna, Veterinärplatz 1, Vienna 1210, Austria; 4 Clinic for Swine, Department for Farm Animals and Veterinary Public Health, University of Veterinary Medicine Vienna, Veterinärplatz 1, Vienna 1210, Austria

## Abstract

Differentiation of porcine T helper cells is still poorly investigated, partly due to a lack of monoclonal antibodies (mAbs) specific for molecules involved in this process. Recently, we identified a mAb specific for porcine CD27 and showed that CD27 is expressed by all naïve CD8α^-^ T helper cells but divides CD8α^+^ T helper cells into a CD27^+^ and a CD27^-^ subset. In the present study, detailed phenotypical and functional analyses of these T-helper cell subpopulations were performed. Naïve CD8α^-^CD27^+^ T helper cells predominantly resided in various lymph nodes, whereas higher proportions of CD8α^+^CD27^+^ and CD8α^+^CD27^-^ T helper cells were found in blood, spleen and liver. Both CD8α^+^CD27^+^ and CD8α^+^CD27^-^ T helper cells were capable of producing IFN-γ upon in vitro polyclonal stimulation and antigen-specific restimulation. Experiments with sorted CD8α^-^CD27^+^, CD8α^+^CD27^+^ and CD8α^+^CD27^-^ T-helper cell subsets following polyclonal stimulation revealed the lowest proliferative response but the highest ability for IFN-γ and TNF-α production in the CD8α^+^CD27^-^ subset. Therefore, these cells resembled terminally differentiated effector memory cells as described in human. This was supported by analyses of CCR7 and CD62L expression. CD8α^+^CD27^-^ T helper cells were mostly CCR7^-^ and had considerably reduced CD62L mRNA levels. In contrast, expression of both homing-receptors was increased on CD8α^+^CD27^+^ T helper cells, which also had a proliferation rate similar to naïve CD8α^-^CD27^+^ T helper cells and showed intermediate levels of cytokine production. Therefore, similar to human, CD8α^+^CD27^+^ T helper cells displayed a phenotype and functional properties of central memory cells.

## Introduction

A peculiarity of porcine T helper cells is the expression of CD8α on a substantial proportion of these cells in blood and secondary lymphatic organs [1,2]. In vitro stimulation by superantigens or mixed leukocyte reactions causes an up-regulation of CD8α expression on porcine T helper cells [1,3], and it was reported that CD8α^+^ T helper cells proliferate in response to stimulation with recall antigen [4-6]. Therefore, CD8α expression is perceived as a marker for activated and memory T helper cells, whereas a CD4^+^CD8α^-^ phenotype is considered to define naïve T helper cells [3]. In addition to CD8α, the expression of CD45RC and swine leukocyte antigen-DR (SLA-DR) was investigated in previous studies to identify different memory stages of CD8α^+^ T helper cells. Differentiation from naïve CD8α^-^ to memory CD8α^+^ T helper cells was described to be accompanied by a loss of CD45RC and an increase in SLA-DR expression [3]. However, an accurate discrimination of functionally distinct T helper cells following antigen contact has remained unsuccessful so far [7].

In human and mouse, differentiation of T helper cells is commonly defined by i) the expression of receptors for lymph node homing, ii) the expression of co-stimulatory molecules and iii) the capability to produce certain cytokines. With regard to the lymph node homing receptors CD62L and CCR7, two functionally distinct memory subsets have been defined: CD62L^+^CCR7^+^ central memory and CD62L^-^CCR7^-^ effector memory T helper cells. Central memory T helper cells proliferate and produce IL-2, whereas effector memory T helper cells secrete high amounts of cytokines such as IFN-γ and IL-4 upon stimulation [8]. Regarding the expression of co-stimulatory molecules, T helper cells initially express CD27, a member of the tumor necrosis factor receptor (TNFR) family, which contributes to proliferation, survival and cytokine production. During T-cell differentiation, CD27 expression undergoes down-regulation and is finally lost on terminally differentiated effector cells [9,10].

In a recent study, we could identify Swine Workshop Cluster 2 as porcine CD27 by the use of a porcine retroviral complementary DNA (cDNA) expression library and the monoclonal antibody (mAb) b30c7 [11]. Regarding the expression of CD27 on porcine T helper cells, it was demonstrated in this study that CD27 is expressed by all naïve CD8α^-^ T helper cells but classifies CD8α^+^ T helper cells into a CD27^+^ and a CD27^-^ subset. Accordingly, due to the presence of CD27^-^ T helper cells only within the CD8α^+^ population, we hypothesized that CD27^+^ and CD27^-^ T helper cells represent separate differentiation stages of porcine T-helper cell development following antigen contact.

Therefore, in the present study, we addressed functional as well as more detailed phenotypical characteristics of CD27-defined T-helper cell subsets in swine. Co-expression of CD4, CD8α, CD27, CD45RC and SLA-DR was analysed within blood, secondary lymphoid organs and liver by flow cytometry (FCM). Functional studies revealed differences in the proliferative capacity and production of the cytokines IFN-γ, TNF-α and IL-2. CD8α^+^CD27^-^ T helper cells showed the lowest proliferation but were superior in IFN-γ and TNF-α release, therefore, resembling effector memory T cells in human. CD8α^+^CD27^+^ T helper cells showed a proliferation similar to the naïve CD8α^-^CD27^+^ fraction and intermediate cytokine production, i.e. attributes reminiscent of central memory T helper cells. This functional allocation was confirmed by phenotypic analyses of the adhesion molecule CD62L as well as the chemokine receptors CCR7 and CX3CR1.

## Materials and methods

### Animals and cell isolation

**Figure 1:**
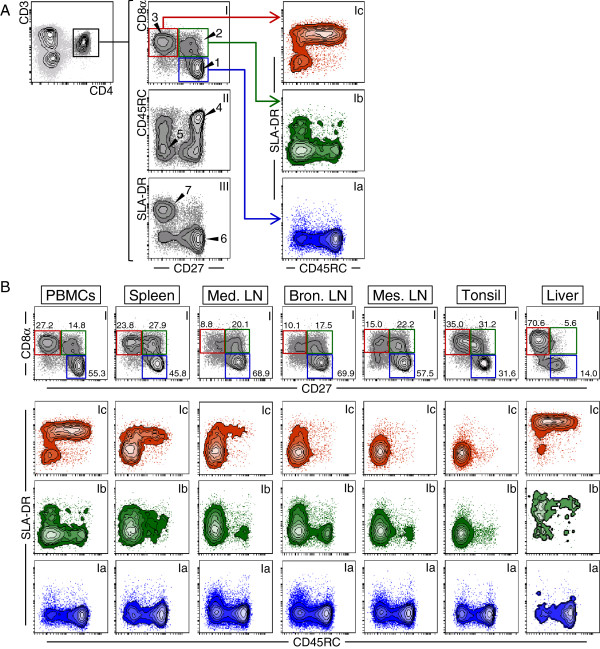
**Phenotype of CD27-defined T-helper cell subsets.** Seven-colour FCM including a live/death discrimination dye was used to study CD45RC and SLA-DR expression on CD8α/CD27-defined T helper cells. (**A**) CD3^+^CD4^+^ T helper cells were gated (upper left contour plot) and analysed for CD27 (x-axis) versus CD8α, CD45RC and SLA-DR (y-axes) expression (middle column). CD8α/CD27-defined cell populations (CD8α^-^CD27^+^, blue gate; CD8α^+^CD27^+^, green gate; CD8α^+^CD27^-^, red gate) were further subgated for CD45RC (x-axes) versus SLA-DR (y-axes) expression as depicted by coloured contour plots (right column). (**B**) CD8α and CD27 expression of CD3^+^CD4^+^ T helper cells as well as CD45RC and SLA-DR expression of CD8α/CD27-defined T-helper cell subpopulations (gating strategy as in A) in blood (PBMCs), spleen, mediastinal (Med. LN), bronchial (Bron. LN) and mesenteric lymph nodes (Mes. LN), tonsil, and liver (columns from left to right; numbers indicate percentage of cells in respective gates). (A + B) See main text for description of arrow heads and numbers. Data of one representative animal out of five is shown. At least 1× 10^5^ lymphocytes per sample were acquired.

**Figure 2:**
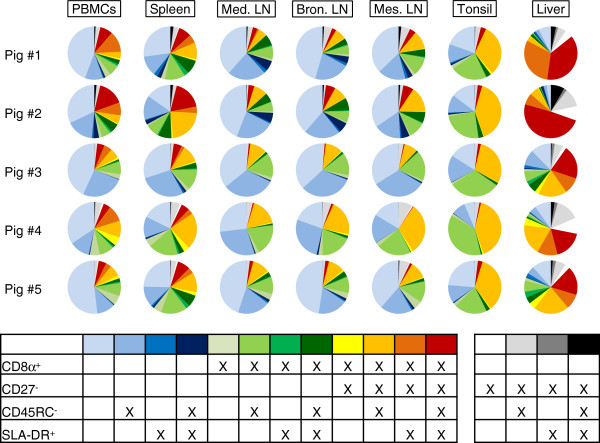
**Combined analysis of CD8α, CD27, CD45RC and SLA-DR expression on T helper cells.** Boolean gating was applied for a linked analysis of the four markers within blood (PBMCs), spleen, mediastinal (Med. LN), bronchial (Bron. LN) and mesenteric lymph nodes (Mes. LN), tonsil, and liver. Results for five animals are shown in pie charts. The table defines the colour code that was used to differentiate the sixteen different subpopulations which result from expression/non-expression of the four markers CD8α, CD27, CD45RC and SLA-DR. Data are derived from FCM analyses described in Figure 1. Only CD3^+^CD4^+^ T helper cells were analysed by boolean gating.

Blood, spleen and mediastinal lymph nodes of healthy six-month old pigs were obtained from an abattoir, where animals were subjected to electric high voltage anaesthesia followed by exsanguination. This procedure is in accordance to the Austrian Animal Welfare Slaughter Regulation. For detailed phenotypical analyses within blood, spleen, mediastinal, bronchial and mesenteric lymph nodes, tonsil and liver (see Figures 1 and 2), samples were obtained from healthy animals kept in-house at the Clinic for Swine of the University of Veterinary Medicine Vienna. Pigs were euthanized at the age of six month by a combination of Ketamine (Narketan®, Vétoquinol GmbH, Vienna, Austria)/Azaperone (Stresnil®, Janssen Pharmaceutica, Beerse, Belgien) anesthesia and T61® (Intervet GmbH, Vienna, Austria).

For studying intracellular IFN-γ production after virus restimulation in vitro, peripheral blood mononuclear cells (PBMCs) were used from animals out of two vaccination/infection experiments. For porcine reproductive and respiratory syndrome virus (PRRSV) restimulation, four-week old piglets were vaccinated intramuscularly with a PRRSV modified live vaccine (Ingelvac® PRRS MLV, Boehringer Ingelheim Vetmedica GmbH, Germany) and challenged intranasally and intramuscularly with a PRRSV field isolate (EU strain, total dose 2.2 × 10^5^ TCID_50_,) 35 days later. PBMCs were isolated on day 21 post challenge.

Moreover, for in vitro restimulation with swine influenza A virus (FLUAVsw), five-week old piglets were intratracheally infected with Influenza A virus/swine/Kitzen/IDT6142/2007 (H1N2, 10^8.25^ TCID_50_ per animal, provided by Dr Ralf Dürrwald, IDT Biologika GmbH, Dessau, Germany). Four weeks later, the piglets were infected for a second time with the same FLUAVsw strain and dose. PBMCs were isolated 35 days after the second infection.

Vaccination/infection experiments with piglets were approved by the institutional ethics committee, the Advisory Committee for Animal Experiments (§12 of Law for Animal Experiments, Tierversuchsgesetz – TVG) and the Federal Ministry for Science and Research (reference numbers BMWF-68.205/0232-II/106/2009 for PRRSV vaccination experiment and BMWF-68.205/0180-II/3b/2011 for FLUAVsw infection experiment).

Heparinized blood was used for isolation of PBMCs by gradient centrifugation with lymphocyte separation medium (PAA Laboratories, Pasching, Austria) as described elsewhere [1]. Cells from spleen, various lymph nodes and tonsil were obtained as reported previously [11]. Isolation of intrahepatic lymphocytes was performed according to a protocol by Crispe [12]. Cells were re-suspended in appropriate buffers (see below) prior to FCM staining. PBMCs used for intracellular cytokine staining and FACS-sorting experiments were cryopreserved at −150°C as described by Leitner et al. [13].

### FCM analyses and antibodies

For phenotypical analysis of CD27-defined T helper cells within PBMCs, spleen, lymph nodes, tonsil and liver, freshly isolated cells were re-suspended in PBS (without Ca^2+^/Mg^2+^, PAA) and 10% (v/v) porcine plasma (in-house preparation) and adjusted to 6 × 10^5^ cells per sample. Samples used to analyse CCR7 expression, were resuspended in PBS (without Ca^2+^/Mg^2+^) containing 10% (v/v) porcine plasma and 10% (v/v) rat serum (PAA). The following primary antibodies were used: CD3-eFluor450 (mIgG1, clone PPT3; custom conjugated to eFluor450 by eBioscience, San Diego, CA, USA), CD4 (mIgG2b, clone 74-12-4), CD8α-PE (mIgG2a, clone 76-2-11; BD Biosciences, San Jose, CA, USA), CD27-Alexa647 (mIgG1, clone b30c7), CD45RC-PerCP-Cy5.5 (mIgG1, clone 3a56), SLA-DR-Qdot605 (mIgG2a, clone MSA3, custom conjugated to Qdot605 by Life Technologies, Carlsbad, CA, USA) and rat anti-human CD197-PE-Cy7 (CCR7) (rat IgG2a, clone 3D12; BD Biosciences). All non-commercial antibodies were produced in-house [14] and – where indicated – purified and covalently conjugated to fluorochromes by the use of either Alexa Fluor647 Monoclonal Antibody Labeling Kit (Life Technologies) or Lightning Link™ PerCP/Cy5.5 conjugation kit (Innova Biosciences, Cambridge, UK) according to manufacturer’s instructions. In a second incubation step, samples were incubated with goat anti-mouse IgG2b-Alexa488 secondary antibody and LIVE/DEAD® Fixable Near-IR dead cell stain (both Life Technologies) as described earlier [11]. For all FCM analyses unspecific binding was evaluated by appropriate isotype-matched control antibodies and compensation settings were calculated based on single-color stained cell samples.

FCM measurements were carried out using a FACSCanto™ II flow cytometer equipped with FACSDiva software (Version 6.1.3., BD Biosciences).

### Intracellular IFN-γ staining

For phorbol 12-myristate 13-acetate (PMA)/Ionomycin stimulation, defrosted PBMCs (2 × 10^5^ per well) were cultured in cell culture medium (RPMI 1640 with stable glutamine supplemented with 10% [v/v] FCS, 100 IU/mL penicillin and 0.1 mg/mL streptomycin (all PAA)) either unstimulated or stimulated with 5 ng/mL PMA and 500 ng/mL Ionomycin (both Sigma-Aldrich, St. Louis, MO, USA) in the presence of 1 μg/mL Brefeldin A (BD GolgiPlug™, BD Biosciences) for 4 h at 37°C.

For virus-specific IFN-γ analyses, PBMCs (2 × 10^5^ per well) from primed animals were in vitro restimulated with either PRRSV or FLUAVsw in cell culture medium with the respective virus by a multiplicity of infection set to 1 (MOI 1). For PRRSV restimulation, the virus strain present in the vaccine was used. For FLUAVsw restimulation, homologous virus as used for the infections was applied. For both viruses, mock-infected micro-cultures served as negative controls. Cells were incubated for 24 h at 37°C. During the last four hours 1 μg/mL Brefeldin A was added to microcultures.

PBMCs were harvested, washed, resuspended in PBS (without Ca^2+^/Mg^2+^) containing 3% (v/v) FCS and finally adjusted to ~2.5 × 10^6^ per sample. In a first incubation step, anti-CD4, anti-CD8α (mIgG2a, clone 11/295/33), and anti-CD27 (clone b30c7) mAbs were added (see above for further details on antibodies). Thereafter, binding of primary antibodies was detected by isotype-matched secondary antibodies: goat anti-mouse IgG1-Alexa488, goat anti-mouse IgG2a-Alexa647 (both Life Technologies) and goat anti-mouse IgG2b-Biotin (Southern Biotech, Birmingham, AL, USA). Free binding sites of secondary antibodies were blocked with whole mouse IgG molecules (2 μg per sample; Jackson ImmunoResearch, West Grove, PA, USA) before cells were incubated with anti-CD45RC-PerCP-Cy5.5 mAb (see above), LIVE/DEAD® Fixable Aqua dead cell stain (Life Technologies) and one of the following streptavidin conjugates: Streptavidin-Qdot605 (Life Technologies, for PMA/Ionomycin stimulation), Streptavidin-Alexa405 (Life Technologies, for PRRSV restimulation) or Streptavidin-eFluor450 (eBiosciences, for FLUAVsw restimulation). Subsequently, cells were fixed and permeabilized as described previously [15] and stained with anti-IFN-γ-PE mAb (mIgG1, clone P2G10; BD Biosciences).

### Fluorescence-activated cell sorting of T-helper cell subsets

For sorting of CD4^+^CD8α^-^CD27^+^, CD4^+^CD8α^+^CD27^+^ and CD4^+^CD8α^+^CD27^-^ T helper cells as well as CD4^-^CD172a^+^ antigen presenting cells (APCs), PBMCs were incubated with primary antibodies against CD8α (clone 11/295/33), CD27 and CD172a (mIgG2b, clone 74-22-15A) [14]. In a second step, binding of primary antibodies was detected with the following isotype-matched secondary antibodies: goat anti-mouse IgG2a-Alexa647 (Life Technologies), goat anti-mouse IgG1-PE and goat anti-mouse IgG2b-Biotin (both Southern Biotech). After blocking free binding sites of secondary antibodies with mouse IgG molecules (Jackson ImmunoResearch), cells were incubated with anti-CD4-FITC mAb (mIgG2b, clone 74-12-4; BD Biosciences) and Streptavidin-Qdot800 conjugate (Life Technologies) in a fourth incubation step. All washing steps were performed with PBS (without Ca^2+^/Mg^2+^) containing 5% (v/v) FCS and 2 mM EDTA (Carl Roth, Karlsruhe, Germany). Finally, cells were subjected to sorting using a FACSAria cell sorter (BD Biosciences) equipped with FACSDiva software (Version 6.1.3., BD Biosciences). The purity of sorted cell populations was 97.6 - 99.9% (mean purity: 99.2%).

### Proliferation assays

For tritium-incorporation assays total PBMCs, CD4^+^CD8α^-^CD27^+^-, CD4^+^CD8α^+^CD27^+^- and CD4^+^CD8α^+^CD27^-^-sorted cells (1.8 × 10^5^ per well) were plated in triplicates and either cultured in cell culture medium alone or stimulated with 5 μg/mL Concanavalin A (ConA) (Amersham Biosciences, Uppsala, Sweden) and 10 IU/mL recombinant human (rh) IL-2 (Roche, Mannheim, Germany). In addition, CD172a^+^ APCs were added to sorted microcultures in a 1:10 ratio. Cells were cultured for 3 days and subsequently pulsed with 1 μCi of ^3^H-thymidine (MP Biomedicals, Eschwege, Germany) per well for additional 18 h. Thereafter, cells were harvested with a Filtermate Harvester (Perkin Elmer, Wellesley, MA, USA). ^3^H-thymidine uptake was quantified in counts per minutes (cpm) by a Top Count 4.00 Scintillation Counter (Perkin Elmer).

For proliferation analyses by FCM, CD4^+^CD8α^-^CD27^+^-, CD4^+^CD8α^+^CD27^+^- and CD4^+^CD8α^+^CD27^-^-sorted cells (5 × 10^6^ cells per sorted fraction) were washed and labelled with CellTrace™ Violet Cell Proliferation Kit (Life Technologies) as described elsewhere [11]. Afterwards, labelled T-helper cell subsets (1.8 × 10^5^ cells per well) were cultivated in presence of CD172a^+^ APCs (2 × 10^4^ cells per well) either in cell culture medium alone or stimulated with 5 μg/mL ConA and 10 IU/mL rhIL-2 for 4 days. Thereafter, cells were washed and resuspended in PBS (without Ca^2+^/Mg^2+^) containing 3% (v/v) FCS. Cell samples were re-stained for CD4, CD8α and CD27 expression by use of anti-CD4 (clone 74-12-4), CD8α and CD27 mAbs. In a second incubation step, the following secondary antibodies were added: goat anti-mouse IgG2b-Alexa488, goat anti-mouse IgG2a-Alexa647 (both Life Technologies) and goat anti-mouse IgG1-PE (Southern Biotech). After two final washes, cells were analysed by FCM. Data was processed by FlowJo software (Version 7.6.3., Tree Star, Ashland, OR, USA).

### Cytokine ELISAs

Supernatants of PBMCs, CD4^+^CD8α^-^CD27^+^-, CD4^+^CD8α^+^CD27^+^- and CD4^+^CD8α^+^CD27^-^-sorted cells stimulated for proliferation analysis (see above) were collected after four days of cultivation. IFN-γ, TNF-α, and IL-2 levels were tested with commercially available porcine IFN-γ ELISA kit (Mabtech, Nacka Strand, Sweden), porcine TNF-alpha DuoSet (R&D Systems, Minneapolis, MN, USA), and Swine IL-2 CytoSet kit (Biosource Europe SA, Nivelles, Belgium), respectively. Optical density was measured at 450/620 nm with a Sunrise ELISA reader (Tecan, Crailsheim, Germany).

### RNA isolation and quantitative RT-PCR (qPCR)

Total RNA was extracted from CD4^+^CD8α^-^CD27^+^-, CD4^+^CD8α^+^CD27^+^- and CD4^+^CD8α^+^CD27^-^-sorted cells using TRI REAGENT™ (Sigma-Aldrich, Vienna, Austria) according to manufacturer’s protocol. RNA quality control and cDNA synthesis were performed as described elsewhere [16]. Aliquots from each cDNA sample investigated in this study were pooled to generate an internal standard (IS), which was used as reference for the quantification.

Primer pairs used to analyse the expression of CD62L and CX3CR1 were newly designed and sequence details are listed in Table 1. Primers (Eurofins MWG Operon, Ebersberg, Germany) were forced to span over exon junctions in order to increase specificity. Conditions for the amplification and validation of the qPCR assays are summarized in Additional file 1. Specificity of the generated PCR products using a cDNA pool was verified by automated sequencing using the pGEM-T Easy vector system (Promega, Madison, WI, USA) and M13 standard sequencing primer (Eurofins MWG Operon).

**Table 1:** Primer pairs used for quantification of gene expression by qPCR

**Target**	**Accession number**	**Primer sequence Forward (F), Reverse (R)**	**Position on + strand**	**Product length (bp)**	**Source**
CD62L	NM_001112678.1	F: AGCAAAGACTCCGGGAAGTG	404	247	designed by primer3 [17]
		R: AGAACTTGCCCAAAGGGTGA	651		
CX3CR1	XM_003358374.1	F: CGCAGGACAGGGTGGCGGAT	−67	217	designed by primer BLAST [18]
		R: ATTGCCCACGAGGCCAAAGGC	150		

Sequences of primers (5’-3’), primer positions on (+) strand relative to the ATG start codon and product length in base pairs (bp) are indicated.

The target genes were analysed using SYBR® green I (×0.5, Sigma) as reporter dye. The qPCR reactions contained iTaq™ polymerase™ (0.3 U/reaction; Bio-Rad), gene specific primers (250 nmol/L each), a final concentration of 200 μmol/L dNTP each and 3 mmol/L MgCl_2_ in the provided reaction buffer (1×). Real-time PCR was performed on a CFX96™ (Bio-Rad, Hercules, CA, USA). The multiplex qPCR assay for the reference genes (β-Actin, Cyclophilin A and GAPDH) was conducted as described previously [19]. Each plate contained corresponding randomly assigned RT-minus controls (30% of all samples investigated), the no-template controls (NTCs) as well as the IS.

Data were analysed using the CFX manager software (Bio-Rad) in the linear regression mode. For the quantification, we applied the method as described elsewhere [19]. The obtained ΔΔCq values of the replicates were averaged and expressed as 2^-ΔΔCq values representing the fold changes relative to IS.

### Statistical analysis

Statistical analyses were performed using SPSS® (IBM, Armonk, NY, USA) Version 20. Means of sorted T-helper cell subsets were proved for normal distribution by the Kolmogorov-Smirnov test. Data sets were subjected to multiple comparisons using one-way analysis of variance with the Bonferroni correction. Two different levels of significance were defined: *p* < 0.05 (indicated by *) and *p* < 0.01 (indicated by **).

## Results

### Phenotypic analyses of porcine T helper cells for CD27 expression in combination with CD8α, CD45RC and SLA-DR

FCM analysis of PBMCs was performed to examine CD27 expression on CD3^+^CD4^+^ T helper cells in combination with three established activation and differentiation markers for swine: CD8α, CD45RC and SLA-DR [3] (Figure 1a, contour plot series “I – III”). Combined analysis of CD27 and CD8α revealed three major subsets: CD8α^-^CD27^+^ (arrow head “1”), CD8α^+^CD27^+^ (arrow head “2”) and CD8α^+^CD27^-^ T helper cells (arrow head “3”). Moreover, CD3^+^CD4^+^ total T helper cells showed a heterogeneous expression for CD45RC within CD27^+^ and CD27^-^ T helper cells, but in tendency the majority of CD27^+^ cells co-expressed CD45RC (arrow head “4”), whereas cells lacking CD45RC slightly dominated within the CD27^-^ population (arrow head “5”). In contrast, within this population of CD3^+^CD4^+^ total T helper cells almost all CD27^+^ cells were SLA-DR^-^ (arrow head “6”), whereas SLA-DR was clearly expressed on the majority of CD27^-^ T helper cells (arrow head “7”).

Analyses of CD8α and CD27 co-expression on CD3^+^CD4^+^ T helper cells were expanded to lymphocytes isolated from secondary lymphatic organs and liver (Figure 1b, contour plot series I). CD8α^-^CD27^+^ T helper cells abounded in lymph nodes (> 57.5%), PBMCs (55.3%) and spleen (45.8%). In contrast, in liver CD8α^+^CD27^-^ T helper cells dominated (70.6%), and the frequency of this population descended from tonsil (35.0%) to PBMCs (27.2%). Of note, in liver, besides few CD8α^-^CD27^+^ and mostly CD8α^+^CD27^-^ T helper cells, a small subset was found that neither expressed CD8α nor CD27 molecules. On the contrary, CD8α^+^CD27^+^ T helper cells were almost entirely absent (5.6%) in liver.

Based on the prevalence of three CD8α/CD27-defined T-helper cell subsets, further phenotypic analyses of CD45RC and SLA-DR expression were attributed to these three subsets (Figure 1a and b; contour plots “I”): CD8α^-^CD27^+^ (blue gate, series “Ia”), CD8α^+^CD27^+^ (green gate, series “Ib”) and CD8α^+^CD27^-^ (red gate, series “Ic”). In PBMCs, secondary lymphatic organs and liver, CD8α^-^CD27^+^ cells contained a major subset of CD45RC^+^ cells and a minor subset of CD45RC^-^ cells which were both SLA-DR^-^ (Figure 1b, contour plot series “Ia”). In contrast, the majority of CD8α^+^CD27^+^ T helper cells were CD45RC^-^ (contour plot series “Ib”). Moreover, except for the tonsil, SLA-DR expression was identified on a subset of CD8α^+^CD27^+^CD45RC^-^ cells. For CD8α^+^CD27^-^ T helper cells (contour plot series “Ic”) within PBMCs and liver, the majority of cells displayed SLA-DR^+^ expression, while CD45RC was heterogeneously expressed. SLA-DR expression was also found on few CD8α^+^CD27^-^ T helper cells obtained from spleen, mediastinal and bronchial lymph nodes and the vast majority of these cells lacked CD45RC expression. In mesenteric lymph node and tonsil nearly all CD8α^+^CD27^-^ T helper cells had a CD45RC^-^SLA-DR^-^ phenotype.

In addition to the hierarchical phenotypic analysis performed in Figure 1, a boolean gating was performed in order to address the complex distribution of phenotypes of T helper cells within various organs (Figure 2). Sixteen distinct CD3^+^CD4^+^ T-helper cell subsets were defined for CD8α, CD27, CD45RC and SLA-DR expression as illustrated by the legend in Figure 2. Based on the expression of CD27 and CD8α, phenotypes of these subsets were clustered into four groups: i) T helper cells with a presumed naïve phenotype, i.e. CD8α^-^CD27^+^ (light blue to dark blue), ii) T helper cells that co-expressed CD27 and CD8α (light green to dark green), iii) T helper cells that were CD8α^+^ but CD27^-^ (yellow to red), and iv) according to results from Figure 1, T helper cells with the rare phenotype CD8α^-^CD27^-^ (white, grey and black). Analyses of five animals revealed large numbers of CD8α^-^CD27^+^SLA-DR^-^ T helper cells which were either CD45RC^+^ or CD45RC^-^ within PBMCs, spleen as well as mediastinal, bronchial and mesenteric lymph nodes (faint blue and light blue, respectively). In tonsils T helper cells predominantly resided with a common CD8α^+^CD45RC^-^SLA-DR^-^ phenotype, but this subset consisted of CD27^+^ and CD27^-^ cells (light green and ochre yellow, respectively). Also, compared to lymph nodes, in PBMCs and spleen more CD8α^+^CD27^-^SLA-DR^+^ cells, which were either CD45RC^+^ or CD45RC^-^, were localized (light red and red, respectively). The proportion of CD27^-^ T-helper cell subsets was highest in liver (ochre yellow, orange and red). Moreover, in liver many CD8α^-^CD27^-^ cells with currently unknown relevance were discovered (white, light grey, black). Overall, the highest diversity of subsets was observed for T-helper cell subsets in PBMCs, spleen and liver.

### IFN-γ production of T-helper cell subsets after polyclonal stimulation and virus-specific restimulation in vitro

**Figure 3:**
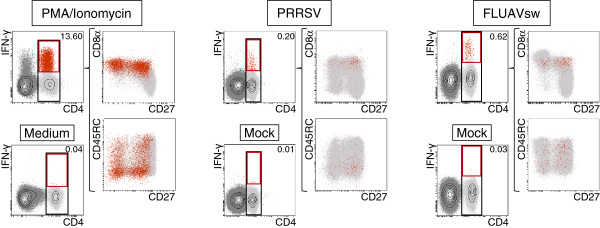
**IFN-γ production of porcine T-helper cell subpopulations.** Intracellular cytokine staining for IFN-γ of PBMCs stimulated with PMA/Ionomycin (left panel), or re-stimulated with PRRSV (middle panel) or FLUAVsw (right panel) was performed by six-colour FCM including a live/death discrimination dye. PBMCs re-stimulated with PRRSV or FLUAVsw were derived from animals that had been vaccinated or infected with the respective virus. Cells cultivated in Medium or Mock served as negative controls. CD4^+^ T helper cells, gated in contour plots (black gates), are shown in dot plots for CD27 (x-axes) versus CD8α and CD45RC expression (y-axes) in light grey. CD4^+^IFN-γ^+^ T helper cells (contour plots; red gates) are highlighted in the respective dot plots in red. Numbers indicate percentage of IFN-γ producing cells within CD4^+^ T helper cells. Data of one representative animal is shown for each stimulation regimen (PMA/Ionomycin: 6 animals tested, PRRSV and FLUAVsw: 3 animals tested for each virus). For PMA/Ionomycin and FLUAVsw at least 3 × 10^5^ lymphocytes and for PRRSV at least 8 × 10^5^ lymphocytes per sample were acquired.

To correlate the phenotype of CD27-defined T-helper cell subsets with functional properties, in a first set of experiments PBMCs were stimulated with PMA/Ionomycin or restimulated with either PRRSV or FLUAVsw. Following stimulation, IFN-γ production of T helper cells was analysed in combination with CD4, CD8α, CD27 and CD45RC expression by FCM (Figure 3). PMA/Ionomycin provoked IFN-γ production in 13.60% of T helper cells. Of note, only CD8α^+^ T helper cells produced IFN-γ. Furthermore, the majority of IFN-γ^+^ T helper cells were CD45RC^-^, whereas both CD27^+^ and CD27^-^ T helper cells were capable of producing IFN-γ. Restimulation with viruses induced much higher levels of IFN-γ producing T helper cells (~20-fold) compared to respective mock stimulated microcultures, thereby demonstrating the specificity of the response. PRRSV induced IFN-γ production in 0.20% and FLUAVsw in 0.62% of T helper cells. Similar to PMA/Ionomycin, PRRSV and FLUAVsw solely stimulated CD8α^+^ T helper cells for IFN-γ release. Again, both viruses caused IFN-γ production of CD27^+^ and CD27^-^ T helper cells, but in tendency more CD27^+^IFN-γ^+^ cells were observed. In regard to CD45RC, for PRRSV most virus-specific IFN-γ^+^ T helper cells were CD45RC^-^, but FLUAVsw evoked more CD45RC^+^ responding cells. In summary, these results indicate that in vivo primed T helper cells are CD8α^+^ but show heterogeneous phenotypes for CD27 and CD45RC.

### Differences in cytokine production and proliferation between CD27-sorted T-helper cell subsets

**Figure 4:**
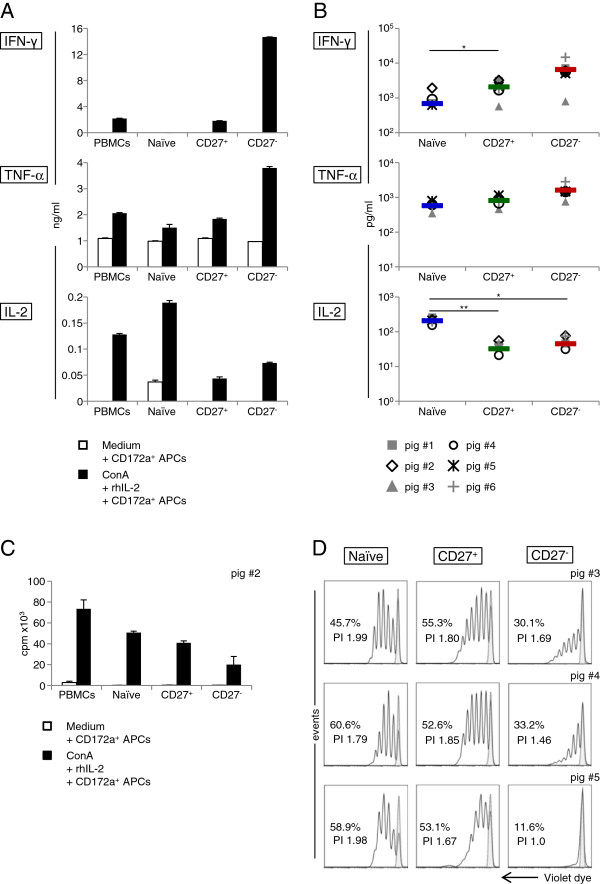
**Cytokine production and proliferation of CD27-sorted T-helper cell subsets.** FACS-sorted CD4^+^CD8α^-^CD27^+^ (naïve), CD4^+^CD8α^+^CD27^+^ (CD27^+^) and CD4^+^CD8α^+^CD27^-^ (CD27^-^) cells were cultured in the presence of 10% CD172a^+^ APCs. Microcultures were cultivated in Medium alone or stimulated with ConA and rhIL-2 for 4 days. (**A**) Supernatants of total PBMCs, naïve, CD27^+^ and CD27^-^-sorted T helper cells were tested for IFN-γ, TNF-α and IL-2 levels by ELISAs. The bar graphs represent mean values + standard deviations of duplicate wells in ng/mL. Representative data of supernatants from one animal out of six is shown. (**B**) Cytokine production of naïve-, CD27^+^- and CD27^-^-sorted T helper cells. Cytokine production in supernatants of cell cultures from six pigs is displayed in pg/mL on a logarithmic scale. The coloured bars indicate the mean values of the six individuals for each T-helper cell subset. Significant differences are denoted (* *p* < 0.05, ** *p<*0.01). (**C**) Total PBMCs, naïve-, CD27^+^- and CD27^-^-sorted T helper cells were analysed for tritium incorporation at day 3 to 4 of cultivation. The bar graph shows counts per minute (cpm) on the y-axis, representing the mean + standard deviation of triplicate cultures. Data is representative for one experiment with cells from one animal out of two. (**D**) Naïve- (left column), CD27^+^- (middle column) and CD27^-^-sorted (right column) T helper cells were labelled with violet proliferation dye prior to cultivation and analysed for proliferation by FCM after four days. Percentage divided and proliferation index (PI) of proliferative cells were determined using FlowJo software and are depicted in the graphs. The dotted lines indicate cells that have not divided as calculated by FlowJo software. Per sample at least 2 × 10^4^ cells were recorded. Data of three animals (top to bottom) are shown.

In a next series of experiments, functional analyses of FACS-sorted CD4^+^CD8α^-^CD27^+^ (now designated as naïve), CD4^+^CD8α^+^CD27^+^ (designated as CD27^+^) and CD4^+^CD8α^+^CD27^-^ (designated as CD27^-^) T-helper cell subsets were performed. Sorted cell fractions were cultivated with ConA and rhIL-2 for 4 days and proliferation as well as production of the cytokines IFN-γ, TNF-α and IL-2 in supernatants was analysed (Figure 4). Results for cytokine production of one representative animal, but also mean values for six individuals are shown in Figure 4a and b, respectively. CD27^-^ T helper cells produced the highest amounts of IFN-γ and TNF-α after 4 days of cultivation (Figure 4a). CD27^+^ T helper cells reached IFN-γ and TNF-α levels similar to unsorted PBMCs. In contrast, the highest detectable IL-2 production was observed for naïve T helper cells. These observations were confirmed by analyses with cells from six individuals (Figure 4b). In addition to cytokine production on day 4, time kinetics of cytokine production for T helper cells from one animal were analysed (Additional file 2). Interestingly, considerable IFN-γ production of CD27^-^ T helper cells was not observed before day 2. In contrast, TNF-α level of CD27^-^ T helper cells was already high on day 1. Naïve T helper cells produced no detectable levels of IL-2 on day 1, but production rapidly increased on day 2.

Proliferation of sorted CD8α/CD27 T-helper cell subsets was analysed by tritium incorporation as well as dilution of violet proliferation dye in response to polyclonal stimulation (Figure 4c and d, respectively). Tritium incorporation revealed that naïve and CD27^+^ T helper cells had similar proliferation capacities, but CD27^-^ T helper cells showed the lowest tritium incorporation (Figure 4c). These findings were also evidenced by violet proliferation assays. Naïve and CD27^+^ T helper cells showed overall similar proliferation indices (PI, average number of cell cycles of responding cells) as well as percentages of divided cells after four days of stimulation (Figure 4d). In opposition to these subsets, CD27^-^ T helper cells clearly demonstrated lowest proliferation rates plus abortive proliferation patterns in two out of three animals tested (pig #3 and #4). The CD27^-^ subset of one animal did not show any proliferation at all (pig #5).

### Phenotypical changes in CD8α/CD27 expression on proliferating T-helper cell subsets

**Figure 5:**
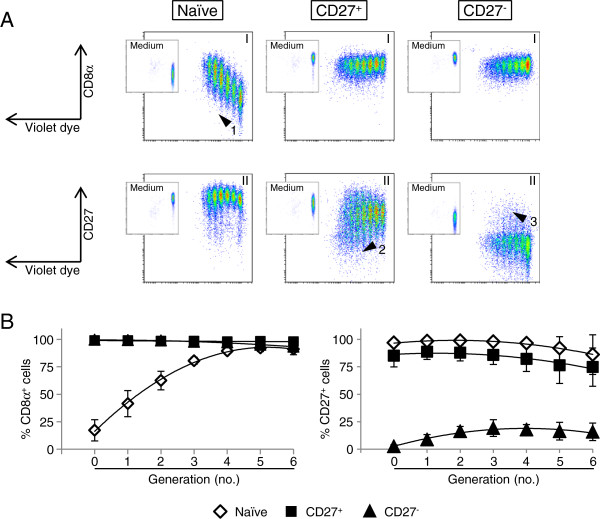
**Changes in CD8α/CD27 expression on proliferating T-helper cell subsets.** (**A**) Violet proliferation dye-labelled CD4^+^CD8α^-^CD27^+^- (left panel; naïve), CD4^+^CD8α^+^CD27^+^- (middle panel; CD27^+^) and CD4^+^CD8α^+^CD27^-^-sorted (right panel, CD27^-^) cells were stimulated with ConA/rhIL-2 in presence of 10% CD172a^+^ APCs for 4 days. Microcultures in Medium served as controls (embedded density plots). Four-colour FCM analysis was performed to analyse Violet-dye dilution (x-axes) versus CD8α and CD27 expression (y-axes) after four days of cultivation. See main text for description of arrow heads and numbers. Data of one representative animal is shown. Per sample at least 2 x 10^4^ cells were recorded. (**B**) Percentage of naïve, CD27^+^ and CD27^-^ sorted T helper cells that expressed CD8α (left graph, y-axis) and CD27 (right graph, y-axis) in relation to cell division (x-axes). Percent values of the respective cell generations were calculated by FlowJo software. The graphs show mean values + standard deviations of three animals tested in separate experiments.

FACS-sorted and violet-labelled T-helper cell subsets were also analysed for potential changes of CD8α and CD27 expression during in vitro cultivation following polyclonal stimulation (Figure 5). On naïve T helper cells CD8α expression was up-regulated on proliferating cells (Figure 5a, arrow head “1”), whereas CD27 expression was barely affected during cultivation. For CD27^+^ and CD27^-^ sorted T-helper cell fractions, CD8α expression remained unaffected. In regard to CD27, a small proportion of CD27^+^ T helper cells seemed to down-regulate the CD27 molecule on the cell surface (Figure 5a, arrow head “2”), and similarly but reverse, a conversion into a CD27^+^ phenotype was observed for a minor fraction of CD27^-^ T helper cells (arrow head “3”). These data were confirmed by a quantitative analysis of the phenotypes in relation to cell generations generated in vitro (Figure 5b). Mean values for CD8α expression on naïve T helper cells from three animals showed a very strong increase of CD8α^+^ cells up to numbers similar to CD27^+^ and CD27^-^ subsets after five or six division cycles. Although naïve T helper cells showed a more stable CD27 expression than CD27^+^ T helper cells, the vast majority of both subsets maintained CD27 expression for 4 days following stimulation, regardless of the number of cell divisions. As described above, a weak increase of CD27^+^ cells was observed within CD27^-^ T helper cells around generation 3.

### Expression of the chemokine-receptors CCR7 and CX3CR1 and the cell adhesion molecule CD62L on CD27-defined T-helper cell subsets

**Figure 6:**
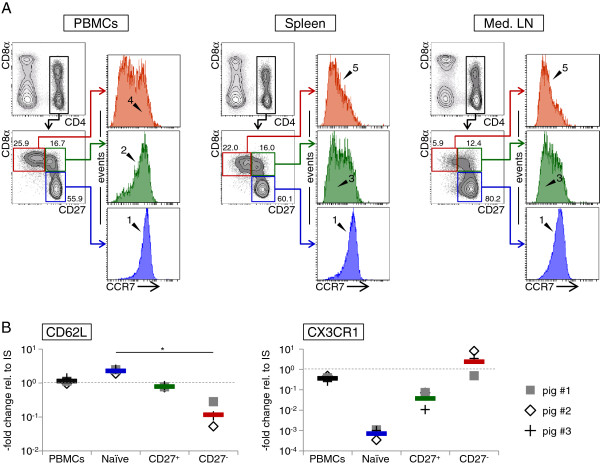
**Expression of CCR7, CD62L and CX3CR1 on CD27-defined T-helper cell subsets.** (**A**) CCR7 expression of T helper cells from blood (PBMCs; left panel), spleen (middle panel) and mediastinal lymph node (Med. LN; right panel) was investigated by five-colour FCM including a live/dead discrimination dye. CD4^+^ T helper cells (upper left contour plots; black gates) were gated and analysed for CD8α (y-axis) and CD27 (x-axis) expression (lower left contour plots). Gates were set on CD8α^-^CD27^+^ (blue gates), CD8α^+^CD27^+^ (green gates) and CD8α^+^CD27 ^-^ (red gates) cell subsets for analyses of CCR7 expression indicated by coloured histograms. Numbers show percentage of gated cell subsets. See main text for description of arrow heads and numbers. Representative data from one individual out of six is shown. Per sample at least 1 × 10^5^ lymphocytes were acquired. (**B**) CD62L and CX3CR1 mRNA expression of total PBMCs and CD27-sorted T-helper cell subsets ex vivo was examined by use of quantitative RT-PCR. The graphs show normalized 2^ΔΔCt values obtained from cells of three animals (symbols) and geometric means (coloured bars) relative to IS. Significant difference is indicated (* *p <* 0.05).

Results described in Figure 4 suggest that CD8α^+^CD27^+^ and CD8α^+^CD27^-^ T helper cells have functional similarities towards central (high proliferation capacity) and effector (rapid production of effector cytokines) memory T cells, respectively, as described in human and mouse [20]. To address this hypothesis, we performed phenotypical analyses for the CD8α/CD27 defined T-helper cell subsets with markers used to identify these memory cell subsets in human, namely, CCR7 and CD62L [8]. Since cross-reactivity of an anti-human CCR7 antibody was recently confirmed for cattle [21] and a specific antibody has not been established for swine so far, we compared the porcine CCR7 amino acid sequence with the sequences from human and cattle. Marked similarities were found, including the binding site of the monoclonal antibody on the N-terminal end of the CCR7 molecule (Additional file 3). Consequently, the human CCR7 antibody was tested and reactivity with porcine lymphocytes could be verified (Figure 6a). CD4^+^ T helper cells isolated from blood, spleen and mediastinal lymph nodes were gated, analysed for CD8α and CD27 expression and CD8α/CD27-defined subpopulations investigated for CCR7 expression. All naïve CD8α^-^CD27^+^ T helper cells expressed CCR7 (arrow heads “1”). Moreover, the vast majority of CD27^+^ T helper cells were CCR7^+^ in PBMCs (arrow head “2”), whereas CCR7^-^ cells became evident in spleen and mediastinal lymph node (arrow heads “3”). For CD27^-^ T helper cells, in blood a mixed population of CCR7^+^ and CCR7^-^ cells was identified (arrow head “4”), but in spleen and mediastinal lymph node CCR7^-^ cells substantially prevailed (arrow heads “5”).

For the adhesion molecule CD62L, quantitative RT-PCR was performed with FACS-sorted CD4^+^CD8α^-^CD27^+^ (naïve), CD4^+^CD8α^+^CD27^+^ (CD27^+^) and CD4^+^CD8α^+^CD27^-^ (CD27^-^) T-helper cell subsets isolated from blood (Figure 6b), since antibodies for this molecule in swine are currently not available. Highest mRNA levels of CD62L were found in naïve T helper cells. CD62L transcript levels were already reduced in CD27^+^ T helper cells and lowest levels were found in CD27^-^ T helper cells. In addition to CD62L, expression of the fractalkine receptor CX3CR1 was analysed at mRNA level (Figure 6b), since CD27^-^ cytotoxic T lymphocytes and T helper cells from human PBMCs were shown to express this molecule [22]. Indeed, in porcine CD27^-^ T helper cells we also found high CX3CR1 mRNA expression, whereas transcripts for this chemokine-receptor were reduced in CD27^+^ T helper cells and lowest levels were found in naïve T helper cells.

## Discussion

During the last decades CD8α, and to a lesser extent, CD29, CD45RA, CD45RC, 2E3 and SLA-DR were analysed as differentiation antigens for the characterization of porcine memory T helper cells [3-6,23-25]. In a model suggested by our group [3], naïve T helper cells were defined by their CD8α^-^CD45RC^+^SLA-DR^-^ expression, whereas memory T helper cells were ascribed to a CD8α^+^CD45RC^-^SLA-DR^+^ phenotype. In a more recent study, we analysed CD27 expression on porcine T and NK cells and identified CD27^-^ cells within activated CD8α^+^ T helper cells. Therefore, we postulated CD27 as a marker for further discrimination of functional T-helper cell subsets in swine [11] since lack of CD27 on human CD4^+^ memory T cells denotes a late stage of differentiation [26,27].

In the present study, we analysed CD27 expression in combination with CD8α, CD45RC and SLA-DR to enable a correlation between older and more recent phenotypic data on porcine T helper cells. Naïve CD8α^-^ T helper cells were mostly CD27^+^ and SLA-DR^-^ but contained a major CD45RC^+^ and a minor CD45RC^-^ subset, most notably in lymph nodes and tonsil. However, in PBMCs and spleen the majority of these cells were CD45RC^+^ (Figure 1b, contour plot series “Ia” and Figure 2, faint blue versus light blue populations). This indicates that the lack of CD45RC expression alone is not a suitable phenotype for the identification of activated or memory porcine T helper cells. Moreover, since CD45RC expression was nearly absent on CD4^+^CD8α^-^ thymocytes (see Additional file 4), our data give hints to speculate that this molecule is up-regulated on naïve T helper cells after maturation in the thymus. Thus, CD8α^-^CD27^+^CD45RC^-^SLA-DR^-^ cells may represent recent thymic emigrants which have been described in human and mouse [28]. This hypothesis is supported by data from a time course study in piglets, where T helper cells from blood were analysed from birth to six month of age. In this study, a small proportion of CD8α^-^CD45RC^-^SLA-DR^-^ T helper cells in blood was already detected at the day of birth [29].

CD8α^+^CD27^+^ T helper cells showed overall a CD45RC^-^SLA-DR^-^ phenotype within blood and secondary lymphatic organs (Figure 1b, contour plot series “Ib”, Figure 2, light green population). By contrast, CD8α^+^CD27^-^ T helper cells demonstrated a very heterogeneous expression for the CD45RC molecule and the majority of these cells were SLA-DR^+^ in blood and liver. However, similar to their CD27^+^ counterparts, CD8α^+^CD27^-^ T helper cells were mostly CD45RC^-^SLA-DR^-^ in secondary lymphatic organs (Figure 1b, contour plot series and “Ic”, Figure 2, ochre yellow population). Therefore, CD8α^+^CD27^-^SLA-DR^+^ T helper cells were most prominent in locations that do not require entry via high endothelial venules but can be accessed by late effector cells. Thus, it is conceivable that SLA-DR expression on CD8α^+^CD27^-^ T helper cells identifies terminally differentiated effector memory cells in swine. For CD45RC expression, a correlation towards differentiation in CD8α^+^ T helper cells is more difficult. In human CD27^-^CD45RA^-^ effector memory T helper cells can re-express CD45RA (CD27^-^CD45RA^+^ T_EMRA_ cells) which show multifunctional T-cell responses to cytomegalovirus infection [30]. Featuring telomere-independent senescence, T_EMRA_ cells especially accumulate in older human patients with persistent viral infections or inflammatory diseases [9,31]. By assuming a similar expression of CD45RA and CD45RC in pigs, one may speculate that CD8α^+^CD27^-^CD45RC^+^ T helper cells represent a T_EMRA_-like population.

In this study, we could also observe a small CD8α^-^CD27^-^ subpopulation of porcine T helper cells which was most prominent in the liver. Considering also the highly reduced frequency of CD8α^+^CD27^+^ cells in this organ, it might be conceivable, that apart from naïve cells present in blood vessels draining the liver, only effector cells with appropriate chemokine receptor and adhesion molecule expression can enter the liver. Therefore, the CD8α^-^CD27^-^ subpopulation might represent exhausted terminal effector cells which down-regulated CD8α expression. In human T cells, CD27 ligation induces a strong up-regulation of anti-apoptotic molecules such as Bcl-xL and Bcl-2 which promote survival of activated effector cells [32,33]. In this context, analyses of anti-apoptotic markers should give further information about distinct T-helper cell subsets in regard to their differentiation stage, including the CD4^+^CD8α^-^CD27^-^ phenotype.

In line with previous data, only CD8α^+^ T helper cells produced IFN-γ after polyclonal stimulation as well as after PRRSV and FLUAVsw restimulation in vitro. Thereby, our study provides further evidence for an activation/memory stage of these cells [3,4,6]. With regard to CD27 expression, PMA/Ionomycin stimulation induced IFN-γ release in both CD27^+^ and CD27^-^ T helper cells. Somewhat in contrast, more CD27^+^ T helper cells responded to recall stimulation with PRRSV and FLUAVsw. In terms of CD45RC expression, we observed high heterogeneity within IFN-γ^+^CD4^+^ T cells for FLUAVsw, whereas more CD45RC^-^ cells responded to PRRSV and PMA/Ionomycin stimulation (Figure 3). In human, CD4^+^ T-cell differentiation can be characterized by expression of markers as CD45RA, CCR7, CD27 and CD28 resulting in a diversity of T-helper cell subsets [9]. Several viral infections such as influenza virus, hepatitis C virus, human immunodeficiency virus, and Epstein-Barr virus infection lead to antigen-specific T helper cells with different phenotypic profiles, but the majority display a central memory phenotype and express CD27 [34-37]. This is in accordance to the IFN-γ^+^CD27^+^ phenotype observed in this study following viral restimulation. Whether this means that CD8α^+^CD27^+^ cells form the major pool of memory cells following FLUAVsw and PRRSV infection or that cells with this phenotype are more adapted to an in vitro restimulation environment, requires further investigations.

Nevertheless, in the present study, we could demonstrate that CD27 expression discriminates three functionally distinct T-helper cell subsets (Figures 4 and 5). In accordance with human data [38], our investigations of sorted CD4^+^ T cells revealed that CD8α^-^CD27^+^ naïve T helper cells produced primarily IL-2 but only reduced amounts of IFN-γ and TNF-α. In consideration of the rapid up-regulation of the CD8α molecule, these cells might have already achieved an early differentiation state at which they were polarized for effector functions, similar to proliferating human naïve CD4^+^ T cells [20,39]. In human T helper cells, the CD27^+^ memory population demonstrates a higher proliferative response than the naïve population [26]. In swine no apparent differences for proliferation between the CD27^+^ naïve and activated subsets were observed. Furthermore, CD8α^+^CD27^+^ T helper cells showed overall an intermediate cytokine production compared to the other subpopulations, thus resembling human central memory T helper cells [8]. In regard to CD27 expression, only few cells down-regulated CD27 after four days of stimulation, although this has been described for human CD27^+^ T helper cells following in vitro stimulation with CD3 and PMA/Ionomycin [27]. One possible explanation is that in vitro a different cytokine milieu prevailed than in vivo and thus differentiation into effector cells did not occur due to the lack of appropriate polarizing cytokines such as IL-12 [20,40]. Similar to human effector memory T helper cells [8], porcine CD27^-^ T helper cells displayed an exhausted proliferation capacity and secreted highest IFN-γ and TNF-α levels.

Analyses of lymph node homing receptors (Figure 6) corroborated our hypothesis that CD8α^+^CD27^+^ cells resemble central memory T helper cells, whereas CD8α^+^CD27^-^ cells are in a more terminally differentiated state and comprise effector memory T helper cells as described for human [8,9]. Expression of CCR7 was confirmed on naïve as well as on the majority of CD8α^+^CD27^+^ T helper cells from blood. Furthermore, both subsets expressed high levels of CD62L mRNA transcripts, thus displaying a tropism towards lymph nodes. On the contrary, CD8α^+^CD27^-^ T helper cells from blood were divided into CCR7^+^ and CCR7^-^ subsets which showed little CD62L mRNA expression, indicating their improved ability to migrate into peripheral tissues. Of note, in human PBMCs, CD27^-^ T helper cells are enriched in the CCR7^-^ population [41] and differentiation of T helper cells is delineated from a CD27^+^CCR7^+^ to a CD27^+^CCR7^-^ and a CD27^-^CCR7^-^ phenotype [9,42]. Similarly, in spleen and lymph nodes, we identified naïve CD8α^-^CCR7^+^ T helper cells and the majority of CD8α^+^ cells were CCR7^-^, regardless of the CD27 phenotype. However, in PBMCs nearly all CD8α^+^CD27^+^ T helper cells expressed CCR7 and only within CD8α^+^CD27^-^ cells a CCR7^-^ population was present. Therefore, although it was only observed for porcine T helper cells from blood, CCR7 might be down-regulated during a later time point of differentiation than CD27.

Lastly, we identified highest mRNA levels of the fractalkine receptor CX3CR1 in the CD8α^+^CD27^-^ T-helper cell subset. This is in accordance with data from human T helper cells, where protein expression of CX3CR1 was found in CD45RA^-^CD27^-^ and CD45RO^+^HLA-DR^+^ T helper cells [22,43].

In conclusion, our data provide novel insights into porcine T-helper cell differentiation. We could demonstrate that CD27 is a useful marker for discrimination of functionally distinct T-helper cell subsets in swine. Overall, the properties of CD27^+^ and CD27^-^ porcine memory T helper cells closely resemble function and phenotype of human central memory and effector memory cells, respectively.

## Abbreviations

APCs: Antigen presenting cells; cDNA: Complementary DNA; ConA: Concanavalin A; FACS: Fluorescence activated cell sorting; FCM: Flow cytometry; FITC: Fluorescein isothiocyanate; FLUAVsw: Swine Influenza A virus; IS: Internal standard; mAbs: Monoclonal antibodies; NTCs: No-template controls; PBMCs: Peripheral blood mononuclear cells; PE: Phycoerythrin; PMA: Phorbol myristate acetate; PRRSV: Porcine reproductive and respiratory syndrome virus; rhIL-2: Recombinant human IL-2; RT-qPCR: Reverse transcription quantitative polymerase chain reaction; SLA-DR: Swine leukocyte antigen-DR; TNFR: Tumor necrosis factor receptor.

## Competing interests

The authors declare that they have no competing interests.

## Authors’ contributions

KR, WG and AS conceived and designed experiments. KR performed experiments. AM, SEE and JCD carried out RT-qPCR analyses. JL and PS helped with additional experiments. AL and MR coordinated the animal experiments. KR and WG wrote the paper. All authors critically revised the manuscript and approved the final version.

## Supplementary Material

Additional file 1 **Optimization and validation of qPCR for CD62L and CX3CR1.** The suitability of the newly designed primers was verified as follows: For CX3CR1 a cDNA pool derived from a set of stimulated (PMA/Ionomycin 6 h, ConA 12 h, and IL2/IL12/IL18 6 h) and unstimulated (6 h, 12 h) total PBMCs was tested in a 1:2 dilution series. For CD62L a 1:10 dilution series of the PCR products was performed. The dilution series, in conjunction with the melt characteristics of the PCR product, were used to optimize the assays regarding the primer concentration as well as the annealing and extension times for the PCR. The resulting slope of the regression analysis corresponding to the efficiency of the qPCR as well as the dynamic range for detecting 100% positive of the lowest dilution are indicated. Calibration curve, melt curve and amplification plot for CD62L and CX3CR1 are depicted. Click here for file

Additional file 2 **Time course study of IFN-γ, TNF-α and IL-2 production in CD27-sorted T helper cells.** Supernatants of FACS-sorted and ConA/rhIL-2 stimulated CD4^+^CD8α^-^CD27^+^ (naïve), CD4^+^CD8α^+^CD27^+^ (CD27^+^) and CD4^+^CD8α^+^CD27^-^ (CD27^-^) cells were collected on day 1, 2 and 4 for ELISAs. The graphs show duration of cultivation on x-axes and mean values for the respective cytokine of duplicate wells in ng/mL on y-axes. Results of one animal are depicted. Click here for file

Additional file 3 **Binding region of anti-CCR7 mAb.** Alignment of full length CCR7 amino acid sequences of human, cattle and pig using GeneDoc Version 2.7.000 [44]. Sequences are derived from Gene Bank by BLAST analyses and homologies are obtained from UniGene (NCBI). Based on the human CCR7 sequence (P32248 [CCR7_HUMAN] reviewed, UniProtKB/Swiss-Prot), the signal peptide (grey), the extracellular (yellow), and cytoplasmic (turquois) domains are highlighted. The red box indicates the binding region of anti-CCR7 mAb 3D12 (BD Biosciences). Click here for file

Additional file 4 **CD45RC expression on thymocytes.** CD45RC expression (histograms) was analysed within four different subpopulations: CD4^-^CD8α^-^, CD4^+^CD8α^+^, CD4^-^CD8α^+^ and CD4^+^CD8α^-^ thymocytes (gates shown on contour plot) by FCM including a live/death discrimination dye. Data of one representative animal out of six is shown. At least 1 × 10^5^ cells per sample were acquired. Click here for file
