# Supplementary material for: CD27 expression discriminates porcine T helper cells with functionally distinct properties
Source: Vet Res. 2013 Mar 11;44(1):18. doi: 10.1186/1297-9716-44-18 (PMC3610194; doi:10.1186/1297-9716-44-18)
Supplement: Additional file 1 — Optimization and validation of qPCR for CD62L and CX3CR1. The suitability of the newly designed primers was verified as follows: For CX3CR1 a cDNA pool derived from a set of stimulated (PMA/Ionomycin 6 h, ConA 12 h, and IL2/IL12/IL18 6 h) and unstimulated (6 h, 12 h) total PBMCs was tested in a 1:2 dilution series. For CD62L a 1:10 dilution series of the PCR products was performed. The dilution series, in conjunction with the melt characteristics of the PCR product, were used to optimize the assays regarding the primer concentration as well as the annealing and extension times for the PCR. The resulting slope of the regression analysis corresponding to the efficiency of the qPCR as well as the dynamic range for detecting 100% positive of the lowest dilution are indicated. Calibration curve, melt curve and amplification plot for CD62L and CX3CR1 are depicted. [file 1297-9716-44-18-S1.pdf]

**Additional file 1** Optimization and validation of qPCR for CD62L and CX3CR1

| Target | Product melt.temp. (°C) | Exon junctions in | Intron size (bp) |
|---|---|---|---|
| CD62L | 88.0 | product | 885/1,816 |
| CX3CR1 | 87.5 | product | 10,756 |

Product melting temperature (°C) and length of intron in base pairs (bp).
Products were spanning over exon junctions.

| Target | Annealing temp./time (°C/s) | Extension temp./time (°C/s) | ΔCt(RT+ to RT-) | Slope | Correlation coefficient (Pearson) $R^2$ | Verified dynamic range |
|---|---|---|---|---|---|---|
| CD62L | 60/30 | 72/20 | >13 | -3.448 | 0.999 | $10^6$ |
| CX3CR1 | 70/30 | 72/30 | >15 | -3.309 | 0.999 | $10^7$ |

Optimized protocol for the amplification of target sequences.

**CD62L** (1:10 serial dilution of PCR product)

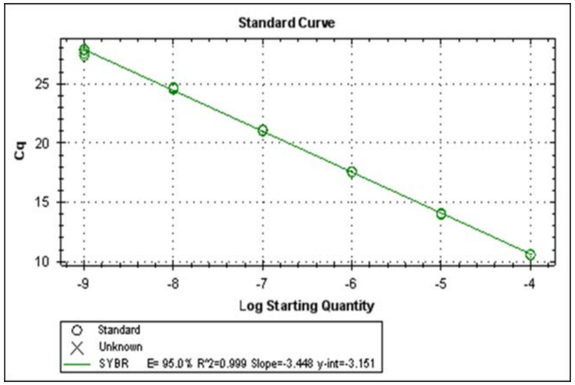


calibration curve

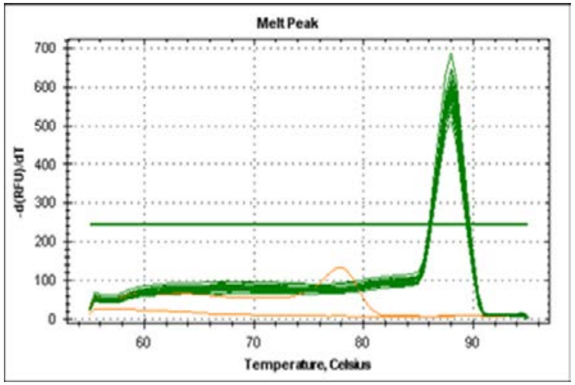


melt curve

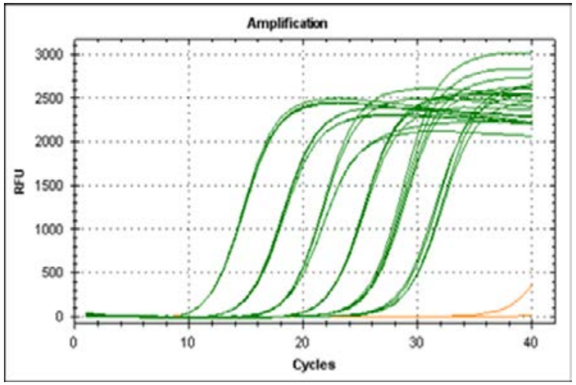


amplification plot

**CX3CR1** (1:2 serial dilution of cDNA pool)

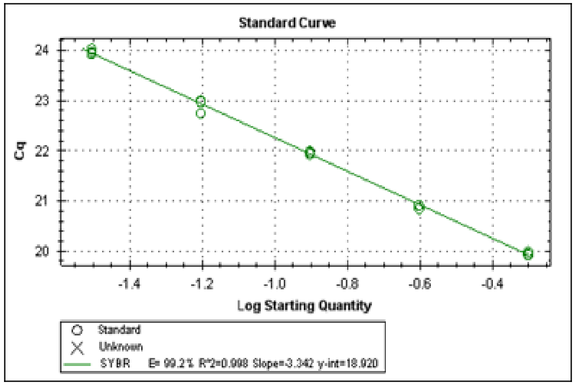


calibration curve

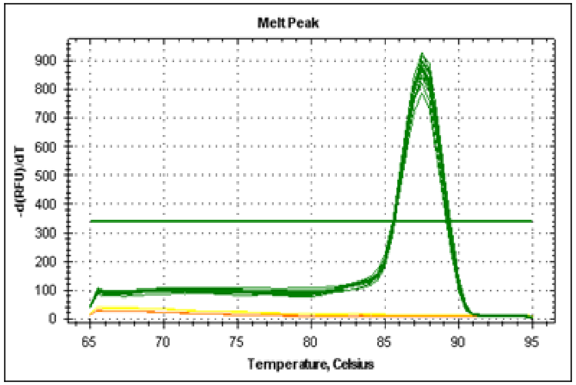


melt curve

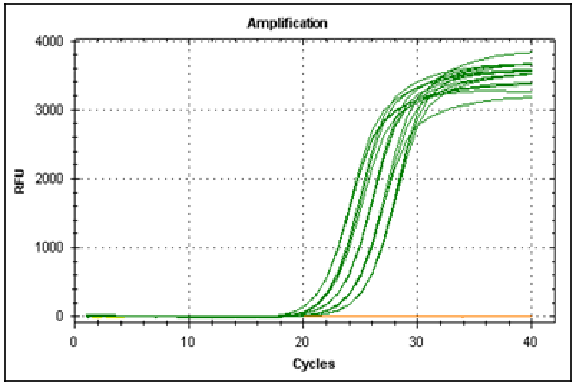


amplification plot
