# Supplementary material for: CD27 expression discriminates porcine T helper cells with functionally distinct properties
Source: Vet Res. 2013 Mar 11;44(1):18. doi: 10.1186/1297-9716-44-18 (PMC3610194; doi:10.1186/1297-9716-44-18)

Additional file 2 Time course study of IFN-γ, TNF-α and IL-2 production in CD27-sorted T helper cells

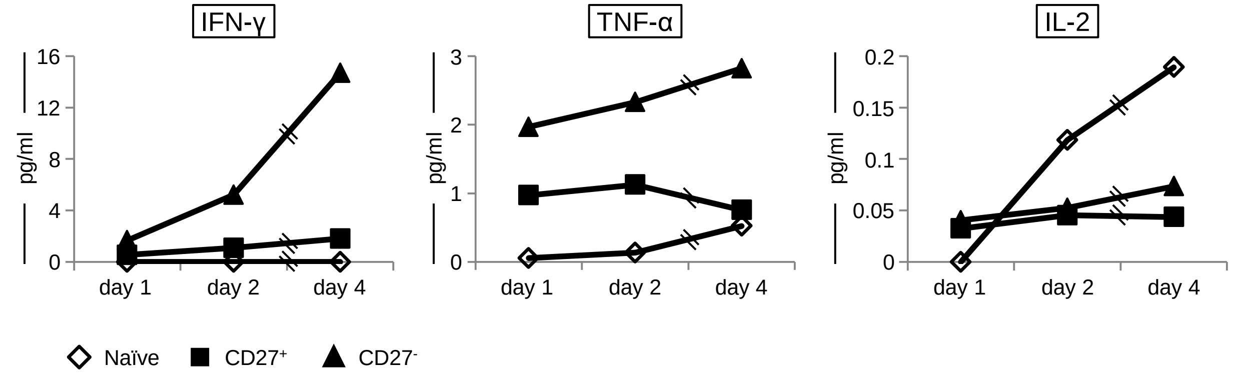

Supplement: Additional file 2 — Time course study of IFN-γ, TNF-α and IL-2 production in CD27-sorted T helper cells. Supernatants of FACS-sorted and ConA/rhIL-2 stimulated CD4+CD8α-CD27+ (naïve), CD4+CD8α+CD27+ (CD27+) and CD4+CD8α+CD27- (CD27-) cells were collected on day 1, 2 and 4 for ELISAs. The graphs show duration of cultivation on x-axes and mean values for the respective cytokine of duplicate wells in ng/mL on y-axes. Results of one animal are depicted. [file 1297-9716-44-18-S2.pdf]
