# Supplementary figures and images for: CD27 expression discriminates porcine T helper cells with functionally distinct properties
Source: Vet Res. 2013 Mar 11;44(1):18. doi: 10.1186/1297-9716-44-18 (PMC3610194; doi:10.1186/1297-9716-44-18)

# Additional file 3 Binding region of anti-CCR7 mAb

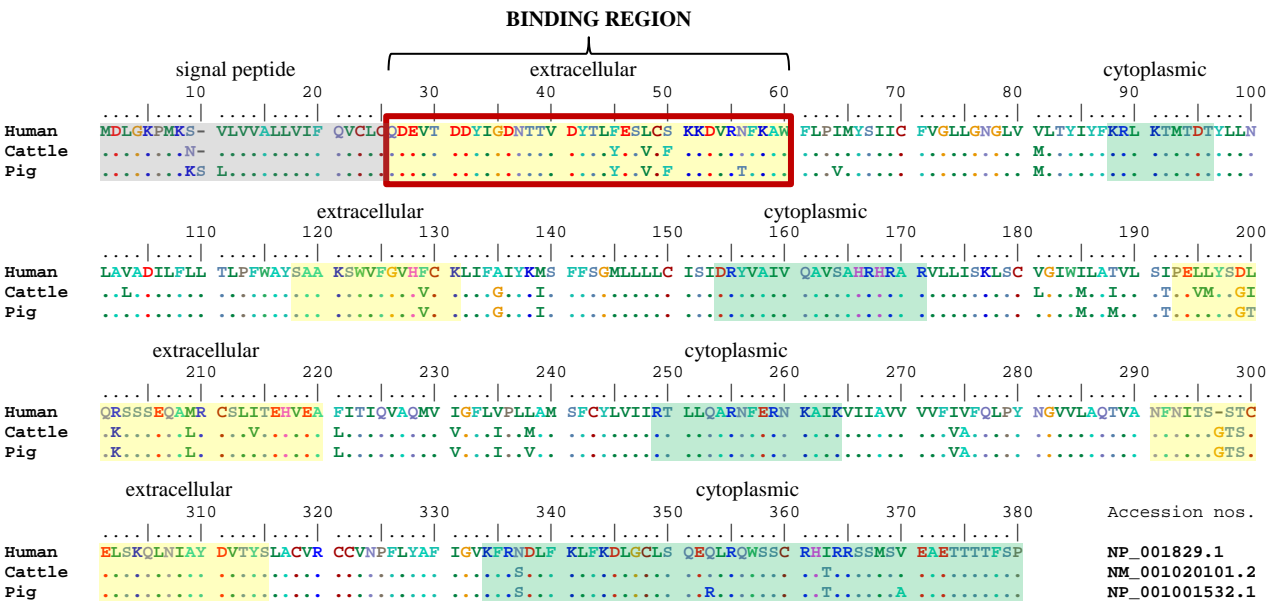

| Species | aa homology (%) | aa length |
|---------|-----------------|-----------|
| Human   | 100             | 378       |
| Cattle  | 95.0            | 379       |
| Pig     | 93.7            | 380       |

Supplement: Additional file 3 — Binding region of anti-CCR7 mAb. Alignment of full length CCR7 amino acid sequences of human, cattle and pig using GeneDoc Version 2.7.000 [44]. Sequences are derived from Gene Bank by BLAST analyses and homologies are obtained from UniGene (NCBI). Based on the human CCR7 sequence (P32248 [CCR7_HUMAN] reviewed, UniProtKB/Swiss-Prot), the signal peptide (grey), the extracellular (yellow), and cytoplasmic (turquois) domains are highlighted. The red box indicates the binding region of anti-CCR7 mAb 3D12 (BD Biosciences). [file 1297-9716-44-18-S3.pdf]

**Additional file 4** CD45RC expression on thymocytes

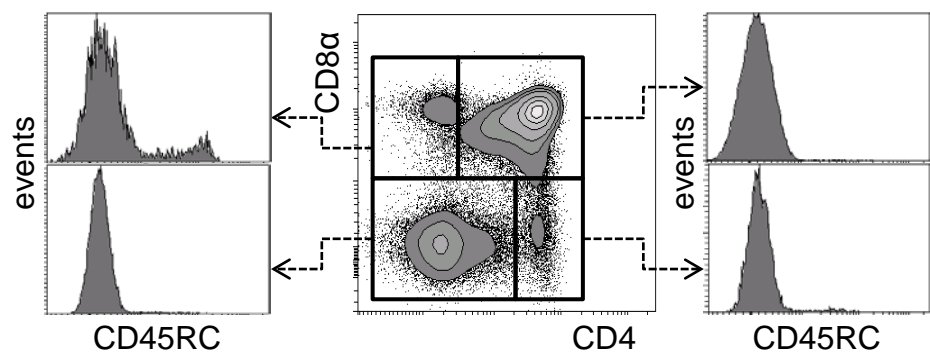

Supplement: Additional file 4 — CD45RC expression on thymocytes. CD45RC expression (histograms) was analysed within four different subpopulations: CD4-CD8α-, CD4+CD8α+, CD4-CD8α+ and CD4+CD8α- thymocytes (gates shown on contour plot) by FCM including a live/death discrimination dye. Data of one representative animal out of six is shown. At least 1 × 10^5^ cells per sample were acquired. [file 1297-9716-44-18-S4.pdf]
